# Smoking in pregnancy: a cross-sectional study in China

**DOI:** 10.1186/s12971-017-0140-0

**Published:** 2017-07-24

**Authors:** Xianglong Xu, Yunshuang Rao, Lianlian Wang, Sheng Liu, Jeff J. Guo, Manoj Sharma, Yong Zhao

**Affiliations:** 1 School of Public Health and Management, Chongqing Medical University, No, 1 Yixueyuan Road, Yuzhong District, Chongqing, 400016 China; 2 Research Center for Medicine and Social Development, Chongqing Medical University, Chongqing, 400016 China; 3 The Innovation Center for Social Risk Governance in Health, Chongqing Medical University, Chongqing, 400016 China; 4 School of Nursing, Chongqing Medical University, Chongqing, 400016 China; 5 The Department of Obstetrics, The First Affiliated Hospital of Chongqing Medical University, Chongqing, 400016 China; 6 Department of Reproduction Health and Infertility, The First Affiliated Hospital of Chongqing Medical University, Chongqing, 400016 China; 7 Canada-China-New Zealand Joint Laboratory of Maternal and Fetal Medicine, Chongqing Medical University, Chongqing, 400016 China; 8 Division of Pharmacy Practice and Administrative Sciences, College of Pharmacy, University of Cincinnati Medical Center, Cincinnati, OH 45267 USA; 9 Department of Behavioral and Environmental Health, Jackson State University, Jackson, 39213 USA

**Keywords:** Smoking, Pregnancy, China

## Abstract

**Background:**

Findings on smoking among pregnant women were mostly from high income countries and were rarely from China. This study aimed to estimate the prevalence of smoking and its influencing factors among pregnant women living in China.

**Methods:**

A cross-sectional analysis was conducted in this study. Data from pregnant women were collected in this study from June to August 2015 from 5 provinces of mainland China. A total of 2345 pregnant women were included in this study, the mean age of the participants was 28.12 years (SD 4.13).

**Results:**

About 82.9% of smoking women quit smoking after they were pregnant. The prevalence of smoking among pregnant women was 3.8%. Among the participants, 40.0, 30.7, 1.8, 29.9, 0.8, 31.4, 31.2, and 26.7% had husbands, fathers-in-law, mothers-in-law, fathers, mothers, colleagues, friends, and relatives, respectively, who were smokers. Compared with pregnant women of basic education level (junior middle school or below), those of the higher education level (undergraduate or above) were at higher risk of smoking (OR, 5.17; 95% CI, 2.00–13.39). Compared with pregnant women from rural areas, urban pregnant women were less likely to be current smokers (OR, 0.55; 95% CI, 0.32–0.94). Compared with pregnant women whose mothers-in-law did not smoke, those whose mothers-in-law smoked were at higher risk of smoking (OR, 4.67; 95% CI, 1.87–11.70). However, compared with pregnant women whose husband did not smoke, those whose husband smoked were not significantly at higher risk of smoking (OR, 1.12; 95% CI, 0.73–1.73).

**Conclusions:**

Most of smoking women quit smoking after they became pregnant. Tailored intervention programs to reduce smoking in pregnant women should focus on those with higher education level, from rural areas, and pregnant women whose mothers-in-law smoke.

## Background

Tobacco use and smoke exposure are critical health issues for pregnant women and their unborn babies [1]. Smoking during pregnancy is significantly associated with increased risks of intrauterine growth retardation [2], low birth weight [3], miscarriage [4], stillbirth [5], congenital malformation [5], early weaning [6], sudden infant death syndrome [7], genetic-related hereditary diseases [8], and childhood overweight [9]. In 2014, 52.9% of men in China were reported to be tobacco smokers, and only 2.4% of the women smoked [10].

Studies in many countries have reported that various factors associated with smoking during pregnancy. These factors include women of relatively young age, of less schooling, who are multiparous, who are exposed to passive smoking (from spouses and friends or colleagues), who have short sleep duration, and who drink [11], mothers born overseas, of higher socio-economic status, pregnant for the first time, and who attended early antenatal care [12]. Further pregnant women who live alone, have low education level (high school or less), have low health literacy, are housewives, have children, have partners who smoke, have unplanned pregnancy, and do not take folic acid [13], have personal stress and complicated personal situation [14], low social support [15], and belong to low income socio-economic status [16] are also associated with greater risk for smoking.

However, findings on smoking among pregnant women were mostly from high income countries and rarely from China. The factors associated with the smoking behavior of pregnant women in China have not been sufficiently clarified. The proximal and distal psychological risk factors of smoking behavior and intervention vary based on the cultural context [17, 18]. Clarifying the factors that affect smoking during pregnancy is necessary to reduce smoking during pregnancy in China, and many other countries with similar socio-cultural norms. This study aims to examine the smoking behavior of pregnant women, as influenced by demographic and socio-economic characteristics and peer influence.

## Methods

### Study design

This multi-city cross-sectional study design and methods have been reported previously [19]. In brief, all the pregnant women visiting 16 hospitals in Chongqing, Chengdu, Zunyi, Liaocheng, and Tianjin, were invited between June and August 2015. Chongqing, Chengdu, and Zunyi are located in south of China; Liaocheng and Tianjin are located in the north of China. Participants were those pregnant women who want to get examined in obstetrics clinic. In total, 2345women participated in the study with a response rate of 97.76% (2400/2455).

### Questionnaire

Demographic data included age (18–25 years old/26–35 years old/36–45 years old), residence (urban/rural), per capita income of the family (<4500¥/4500¥ to 9000 ¥/>9000¥), only child (Yes/No), husband is the only child (Yes/ No), marital status (unmarried / married/ remarried/ divorced or widowed), occupation (rural migrant workers/urban and rural unemployed, unemployed/ Industrial workers of non-agricultural registered permanent residence/individual business/business services staff/ civil servants/ senior manager and middle-level manager in large and medium enterprise/ private entrepreneur/ professionals/ clerks/ students/ others), and hospital level (Level 3A hospital/Level 2 A hospitals/Level 2B hospitals and below), nationality (Han nationality/Minority). Pregnancy was divided into three trimesters. Education level was categorized as ≤ primary school, junior middle school (basic education), ≥ senior high school (including vocational/technical secondary school and junior college), secondary education, and ≥ senior college and university (higher education).

In the multivariate analysis for factors that affect smoking among pregnant women, employment status was categorized as non-manual (individual business/civil servants/senior manager and middle-level manager in large and medium enterprise/private entrepreneur/professionals/clerk/students), manual (rural migrant workers/industrial workers of non-agricultural registered permanent residence/business services staff), unemployed, and others [20].

### Smoking status

Husband, father-in-law, mother-in-law, father, mother, colleague, friend, relative were divided into smokers and non-smokers. Colleague (at least one person defined as a smoker); Friend (at least one person defined as a smoker); Relative (at least one person defined as a smoker); currently smoking (defined as those who smoke during pregnancy).

### Statistical analyses

The characteristics of the participants were summarized using frequencies and percentages and presented using descriptive analysis. Univariate logistic regression analysis and multivariable logistic regression analysis was utilized to probe factors affecting smoking among pregnant women. Factors were considered in the multivariate logistic regression modeling of factors that affect smoking among pregnant women: parity, education level, residence, career, trimester of pregnancy, smoking status of husband, smoking status of father-in-law, and smoking status of mother-in-law. Multivariate model was statistically significant in the model coefficient test (*p* < 0.05), and reached a good fit in Hosmer and Lemeshow test (*p* = 0.9688). All statistics were performed using a two-sided test, and statistical significance was considered at *p* < 0.05. Data analyses were performed using statistical software (SAS version 9.1.3; SAS Institute, Cary, NC, USA).

## Results

### Characteristics of participants

A total of 2345 pregnant women were included in this study, including 1755 [74.84%] pregnant women of first pregnancy. The mean age of the participants was 28.12 years (SD 4.13). 164 (6.99%) women smoked prior to pregnancy among those smokers, 136(82.9%) quitted smoking after they were pregnant. Overall, the prevalence of smoking was 90 [3.8%] (74.4 and 25.6% women of first and second pregnancies, respectively). (Please see Table 1).

**Table 1:** Characteristics of pregnant women, China, 2015 (n, %)

Variable	All participants	Smoker	Non-smoker
Hospital level			
Level 3A hospital	1824(77.8)	1752(77.7)	72(80.0)
Level 2 A hospitals	311(13.3)	301(13.4)	10(11.1)
Level 2B hospitals and below	210(9.0)	202(8.9)	8(8.9)
Parity			
Pregnant women in their first pregnancy	1755(74.8)	67(74.4)	1688(74.9)
Pregnant women s in their second pregnancy	590(25.2)	23(25.6)	567(25.1)
Nationality			
Han nationality	2252(96.0)	88(97. 8)	2164(96.0)
Minority	93(4.0)	2(2.2)	91(4.0)
Single-child			
Yes	1046(44.6)	36(40.0)	1010(44.8)
No	1299(55.4)	54(60.0)	1245(55.2)
Husband was single-child			
Yes	1173(50.0)	43(47.8)	1130(50.1)
No	1172(50.0)	47(52.2)	1125(49.9)
Marital status			
Unmarried	49(2.1)	4(4.4)	45(2.0)
Married	2205(94.0)	81(90.0)	2124(94.2)
Remarried	70(3.0)	4(4.4)	66(2.9)
Divorced or Widowed	21(0.9)	1(1.2)	20(0.9)
Education level			
Basic education	402(17.1)	5(5.6)	397(17.6)
Secondary education	354(15.1)	11(12.2)	343(15.2)
Higher education	1589(67.8)	74(82.2)	1515(67.2)
Residence			
Urban	1880(80.2)	69(76.7)	1811(80.3)
Rural	465(19.8)	21(23.3)	444(19.7)
The per capita income of the family			
< 4500¥	611(26.0)	25(27.8)	586(26.0)
4500¥ to 9000 ¥	989(42.2)	33(36.7)	956(42.4)
> 9000¥	745(31.8)	32(35.5)	713(31.6)
Trimester of pregnancy			
First trimester	293(12.5)	14(15.6)	279(12.4)
Second trimester	701(29.9)	30(33.3)	671(29.8)
Third trimester	1351(57.6)	46(51.1)	1305(57.8)
Age			
18–25 years old	624 (26.6)	19(21.1)	605(26.8)
26–35 years old	1595(68.0)	67(74.4)	1528(67.8)
36–45 years old	126(5.4)	4(4.5)	122(5.4)
Occupation			
Rural migrant workers	118(5.0)	2(2.2)	116(5.1)
Urban and rural unemployed	553(23.6)	18(20.0)	535(23.7)
Industrial workers of Non-agricultural registered permanent residence	50(2.1)	3(3.3)	47(2.1)
Individual business	199(8.5)	9(10.0)	190(8.4)
Business services staff	155(6.6)	10(11.1)	145(6.4)
Civil servants	398(17.0)	16(17.8)	382(17.0)
Senior manager and Middle-level manager in large and medium enterprise	96(4.1)	4(4.5)	92(4.1)
Private entrepreneur	87(3.7)	2(2.2)	85(3.8)
Professionals	244(10.4)	9(10.0)	235(10.4)
Clerk	139(5.9)	5(5.6)	134(5.9)
Students	15(0.6)	0(0.0)	15(0.7)
Other	291(12.4)	12(13.3)	279(12.4)
Smoking status of peers of pregnant women			
Husband			
Smoker	939(40.0)	38(42.2)	901(40.0)
Non-smoker	1406(60.0)	52(57.8)	1354(60.0)
Father-in-law			
Smoker	719(30.7)	26(28.9)	693(30.7)
Non-smoker	1626(69.3)	64(71.1)	1562(69.3)
Mother-in-law			
Smoker	42(1.8)	6(0.3)	36(1.6)
Non-smoker	2303(98.2)	36(1.6)	2219(98.4)
Father			
Smoker	700(29.8)	27(30)	673(29.8)
Non-smoker	1645(70.2)	63(70)	1582(70.2)
Mother			
Smoker	18(0.8)	1(1.1)	17(0.7)
Non-smoker	2327(99.2)	89(98.9)	2238(99.3)
Colleague			
Smoker	736(31.4)	24(26.7)	712(31.6)
Non-smoker	1609(68.6)	66(73.3)	1543(68.4)
Friend			
Smoker	732(31.2)	26(28.9)	706(31.3)
Non-smoker	1613(68.8)	64(71.1)	1549(68.7)
Relative			
Smoker	625(26.6)	21(23.3)	604(26.8)
Non-smoker	1720(73.4)	69(76.7)	1651(73.2)

### Univariate logistic regression model for identifying factors that affect smoking in pregnancy

Univariate logistic regression model for identifying factors affect smoking among pregnant women (Please see Table 2). Compared with pregnant women of basic education level (junior middle school or below), those of higher education level (senior college and university or above) were at higher risk of smoking (OR, 3.88; 95% CI, 1.56–9.66). Compared with pregnant women whose mothers-in-law did not smoke, those whose mothers-in-law smoked were at higher risk of smoking (OR, 4.40; 95% CI, 1.81–10.74).

**Table 2:** Univariate logistic regression analysis for factors that affect smoking among pregnant women, China, 2015

Variable	OR(95%CI)	*p*-value
Hospital level		
Level 2 A hospitals vs. Level 2B hospitals and below	1.04(0.49,2.19)	0.923
Level 3A hospital vs. Level 2B hospitals and below	0.84(0.33,2.16)	0.716
Parity		
Pregnant women in their second pregnancy vs. Pregnant women in their first pregnancy	1.02(0.63,1.66)	0.929
Nationality		
Minority vs. Han nationality	0.54(0.13,2.23)	0.395
Single-child		
Yes vs. No	0.82(0.54,1.26)	0.371
Husband was single-child		
Yes vs. No	0.91(0.60,1.39)	0.664
Marital status		
Unmarried vs. Married	2.33(0.82,6.64)	0.112
Remarried vs. Married	1.59(0.57,4.47)	0.380
Divorced or Widowed vs. Married	1.31(0.17,9.89)	0.793
Education level		
Secondary education vs. Basic education	2.55(0.88,7.40)	0.086
Higher education vs. Basic education	3.88(1.56,9.66)	0.004
Residence		
Urban vs. Rural	0.81(0.49,1.33)	0.396
The per capita income of the family		
4500¥ and 9000 ¥ vs. <4500¥	0.81(0.48,1.37)	0.433
> 9000¥ vs. <4500¥	1.05(0.62,1.80)	0.853
Trimester of pregnancy		
Second trimester vs. First trimester	0.89(0.47,1.71)	0.727
Third trimester vs. First trimester	0.70(0.38,1.30)	0.258
Age		
26–35 years old vs. 18–25 years old	1.40(0.83,2.34)	0.207
36–45 years old vs. 18–25 years old	1.04(0.35,3.12)	0.939
Occupation		
Manual vs. Non-manual	1.23(0.68,2.23)	0.503
Unemployed vs. Non-manual	0.85(0.49,1.48)	0.559
Others vs. Non-manual	1.08(0.57,2.08)	0.810
Smoking status of peers of pregnant women		
Husband (Smoker vs. Non-smoker)	1.10(0.72,1.68)	0.667
Father-in-law (Smoker vs. Non-smoker)	0.92(0.58,1.46)	0.710
Mother-in-law (Smoker vs. Non-smoker)	4.40(1.81,10.74)	0.001
Father (Smoker vs. Non-smoker)	1.01(0.64,1.60)	0.975
Mother (Smoker vs. Non-smoker)	1.48(0.20,11.24)	0.705
Colleague (Smoker vs. Non-smoker)	0.78(0.49,1.27)	0.326
Friend (Smoker vs. Non-smoker)	0.89(0.56,1.42)	0.627
Relative (Smoker vs. Non-smoker)	0.83(0.51,1.37)	0.468

### Multivariable logistic regression model for identifying factors that affect smoking among pregnant women

Multivariate logistic regression model for identifying factors affect smoking among pregnant women (Please see Table 3). Compared with pregnant women of basic education level (junior middle school or below), those of higher education level (senior college and university or above) were at higher risk of smoking (OR, 5.17; 95% CI, 2.00–13.39). Compared with pregnant women from rural areas, urban pregnant women were less likely to be current smokers (OR, 0.55; 95% CI, 0.32–0.94). Compared with pregnant women whose mothers-in-law did not smoke, those whose mothers-in-law smoked were at higher risk of smoking (OR, 4.67; 95% CI, 1.87–11.70). However, compared with pregnant women whose husband did not smoke, those whose husband smoked were not significantly in higher risk of smoking (OR, 1.12; 95% CI, 0.73, 1.73). Pregnant women in their second pregnancy were not significantly in the prevalence of smoking among pregnant women than those in their first pregnancy (OR = 1.19, 95% CI = 0.73, 1.96).

**Table 3:** Odds ratio (95%CI) for identifying factors that affect smoking among pregnant women, China, 2015

Parameter	OR (95% CI)	*p*-value
Parity		
Pregnant women in their second pregnancy vs. Pregnant women in their first pregnancy	1.19(0.73,1.96)	0.482
Education level		
Secondary education vs. Basic education	2.69(0.92,7.89)	0.071
Higher education vs. Basic education	5.17(2.00,13.39)	0.001
Residence		
Urban vs. Rural	0.55(0.32,0.94)	0.028
Smoking status of Husband		
Smoker vs. Non-smoker	1.12(0.73,1.73)	0.606
Smoking status of Father-in-law		
Smoker vs. Non-smoker	0.88(0.54,1.42)	0.598
Smoking status of Mother-in-law		
Smoker vs. Non-smoker	4.67(1.87,11.70)	0.001

*Note*: (1) Adjusted OR was adjusted for Parity, Education level, Residence, Smoking status of Husband, Smoking status of Father-in-law, Smoking status of Mother-in-law; (2) *Abbreviation*: *CI* confidence intervals, *OR*: odds ratio

## Discussion

The family planning policy of China was introduced in 1979 to slow the population growth rate of the nation. At the end of 2013, “Selective Two-Child Policy” was introduced China’s amendment to its 1978 single-child family policy, and it allowed couples nationwide to have a second child if either parent is an only child. Then, on 29 October 2015, the Chinese government announced the “2nd-child policy”, fully enabling couples to have two children. This means that more pregnant women and pregnant women in their second pregnancy in the future. Health behavior and health during pregnancy are worthy of attention. Smoking behavior is a critical health issue for pregnant women. This study found that the prevalence of smoking was 3.8%, which was higher than the smoking rate of women in general (2.4%) [21]. Possible reason is that pregnant women in this study of higher education level are more likely to smoke, and 67.8% of participants in this study were in higher education level. Although a study found that first time mothers showed an increased likelihood of smoking cessation during pregnancy [12], this study found second time mothers were not significantly correlated with low smoking during pregnancy in this study.

This study found that pregnant women of the higher education level (senior college and university or above) were at higher risk of smoking than those of the basic education level. This study is different from the study in Japan, in which the prevalence of smoking was significantly higher among pregnant women with less schooling [11]. Education was different because of the different social systems and cultures. We hypothesize the possible reason to be that persons with high education level do not necessarily have high health literacy and health knowledge levels. Most Chinese with higher education level focus on their own professional learning, and they may acquire little health literacy and health knowledge. A previous study showed that no statistically significant correlations existed between smoking cognition and behavior among young male smokers in a sample with higher education in China [22]. People with high levels of health literacy are more likely to engage in health-promoting behaviors and therefore have better health [23]. Another possible reason is that most women with higher education level have jobs, and they may be affected by the environment, for example, the male colleague smokers. The reason behind that the high proportion of smoking rate among pregnant women of higher education level remains undetermined, and further research will be needed to probe the reasons.

This study found that compared with rural pregnant women, urban pregnant women were less likely to be current smokers. We hypothesize the possible reason to be that rural pregnant women are more likely to be lack of antenatal care than urban pregnant women. A previous study found that lack of antenatal care in the first trimester was strongly associated with increased risk of smoking during pregnancy [12]. Secondly, smoking culture is widely popular in rural areas, more than half of them live in rural areas in China [24], and smoking prevalence in rural areas was higher than in urban areas [25]. Among current and former smokers, sharing cigarettes in China was a major impediment to smoking cessation [26]. In addition, this may be related to the difference between rural and urban settings on the maternity care during pregnancy in China, such as maternity care institutions, the quality of hospital, health-rated information and health education. Persons in rural areas have limited access to health education information. Consequently, rural residents have low level of health literacy. A previous survey showed that the scientific literacy level of urban residents in 2015 was 9.72%, which was higher than that of rural residents (2.43%) [27]. Compared with health education activities in urban areas, rural areas have limited health education activities. Thus, pregnant women in rural areas may be unaware of the harm of smoking during pregnancy. Consequently, the active promotion of antismoking education in rural, poverty-stricken, and less developed areas is important. This study may indicate that strengthening the constructions of rural popular science of public service and scientific information is necessary.

This study found that pregnant women whose mothers-in-law smoked were at higher risk of smoking than those whose mothers-in-law did not smoke. However, this study also found that smoking status of husband and smoking status of father-in-law did not significantly with the risk of smoking among pregnant women. Smoking behavior by social network members increases the likelihood of smoking, and this effect appears to generalize to pregnant women in China. We hypothesize the possible reason to be that most women in China live with their husband’s family and not with their parents after marriage. Married women may have more contact with their husband’s parents. Thus, some behavior or habits of pregnant women are more susceptible to the influence of mothers-in-law. The positions of mothers-in-law in their home are higher in the eyes of pregnant women, thus the impact on pregnant women in terms of smoking. Although the rates of smoking among husbands are high, the influence of peers and husband is small and not statistically significant in China, which is different from that in Western population. Studies found that peer influence (husband, colleagues, and relatives) has a role in the smoking of pregnant woman [2, 28]. Our study found that pregnant women smokers received more pressure from mothers-in-law than from husbands, fathers-in-law, fathers, mothers, colleagues, friends, and relatives. Proximal factors include peer influence, which is often expressed as peer pressure [29]. The proximal and distal psychological risk factors of smoking behavior and intervention vary based on the cultural context [28]. This study provides some directions and insights for future health education on tobacco control among pregnant women. Health educators not only need to focus on pregnant women but also on mothers-in-law of pregnant women in future health education on smoking, especially for pregnant women.

This study has certain limitations. First, cross-sectional survey data reduced the ability to make direct causal inferences, explore whether unmeasured factors may better explain the observed relationships we observed, and determine the direction of causality. Second, the face-to-face interview survey may have resulted in information bias. Some smoking respondents may not have answered the questions truthfully. Some smokers may report that they were non-smokers, and this could underestimate the prevalence of smoking among pregnant women. However, all questions in the survey were reviewed by a research panel and the participants in the pilot study. Thus, the questionnaire was less likely to have included items that could be perceived as sensitive by the study participants. Third, our study was not exactly nationally representative. The sample consisted of pregnant women in five regions of China, namely, Chongqing, Chengdu, Zunyi, Liaocheng, and Tianjin. Chongqing, Chengdu, and Zunyi are in south China, whereas Liaocheng and Tianjin are in north China. Fourth, we did not compare the effect of different numbers of smokers in the three groups (colleagues, friends, and relatives) on smoking pregnant women in this study because calculating the number of smokers in the three groups is very difficult.

## Conclusions

Most of smoking women quit smoking after they became pregnant. Tailored intervention program to reduce smoking in pregnant women should focus on pregnant women with higher education level, from rural areas, and whose mothers-in-law smoke. Health education workers need to consider these factors fully in future planning to help pregnant women smokers quit smoking. These findings have implications for the WHO recommendations on prevention and management of tobacco use in pregnancy, especially for China.
